# Exosomal noncoding RNAs in Glioma: biological functions and potential clinical applications

**DOI:** 10.1186/s12943-020-01189-3

**Published:** 2020-03-25

**Authors:** Jian Cheng, Jinli Meng, Lei Zhu, Yong Peng

**Affiliations:** 1 Laboratory of Molecular Oncology, State Key Laboratory of Biotherapy and Cancer Center, West China Hospital, Sichuan University, Chengdu, 610041 China; 2 Department of Neurosurgery, West China Hospital, Sichuan University, Chengdu, China; 3 Department of Radiology, Hospital of Chengdu Office of People’s Government of Tibetan Autonomous Region (Hospital C.T.), Chengdu, China

**Keywords:** Exosome, Extracellular vesicles, Noncoding RNA, Glioma, Cancer diagnosis, Cancer therapy

## Abstract

Gliomas are complex and heterogeneous brain tumors with poor prognosis. Glioma cells can communicate with their surroundings to create a tumor-permissive microenvironment. Exosomes represent a new means of intercellular communication by delivering various bioactive molecules, including proteins, lipids and nucleic acids, and participate in tumor initiation and progression. Noncoding RNAs (ncRNAs) including microRNA, long-noncoding RNA, and circular RNA, account for a large portion of human transcriptome and play important roles in various pathophysiological processes, especially in cancers. In addition, ncRNAs can be selectively packaged, secreted and transferred between cells in exosomes and modulate numerous hallmarks of glioma, such as proliferation, invasion, angiogenesis, immune-escape, and treatment resistance. Hence, the strategies of specifically targeting exosomal ncRNAs could be attractive therapeutic options. Exosomes are able to cross the blood brain barrier (BBB), and are readily accessible in nearly all types of human biofluids, which make them the promising biomarkers for gliomas. Additionally, given the biocompatibility of exosomes, they can be engineered to deliver therapeutic factors, such as RNA, proteins and drugs, to target cells for therapeutic applications. Here, we reviewed current research on the roles of exosomal ncRNAs in glioma progression. We also discussed their potential clinical applications as novel biomarkers and therapeutics.

## Introduction

Glioma is the most common and malignant primary tumor of the central nervous system (CNS). Glioblastoma (GBM), the most lethal glioma, accounts for 70% of all diffuse glioma diagnoses and has a median overall survival of 15 months [1]. Currently, standard GBM treatments include maximal safe surgical resection and combined radio-chemotherapy [2]. Apart from the rapid proliferation, extensive invasion, intra- and inter-tumoral genetic heterogeneity, and treatment resistance of GBM, the dismal prognosis of GBM patients also originates from poor understanding of molecular pathogenesis and lacking of timely diagnosis and sensitive therapeutic monitoring tools [3]. Therefore, it is crucial to elucidate molecular mechanisms underlying glioma development and progression, and further explore reliable biomarkers.

**Fig. 1:**
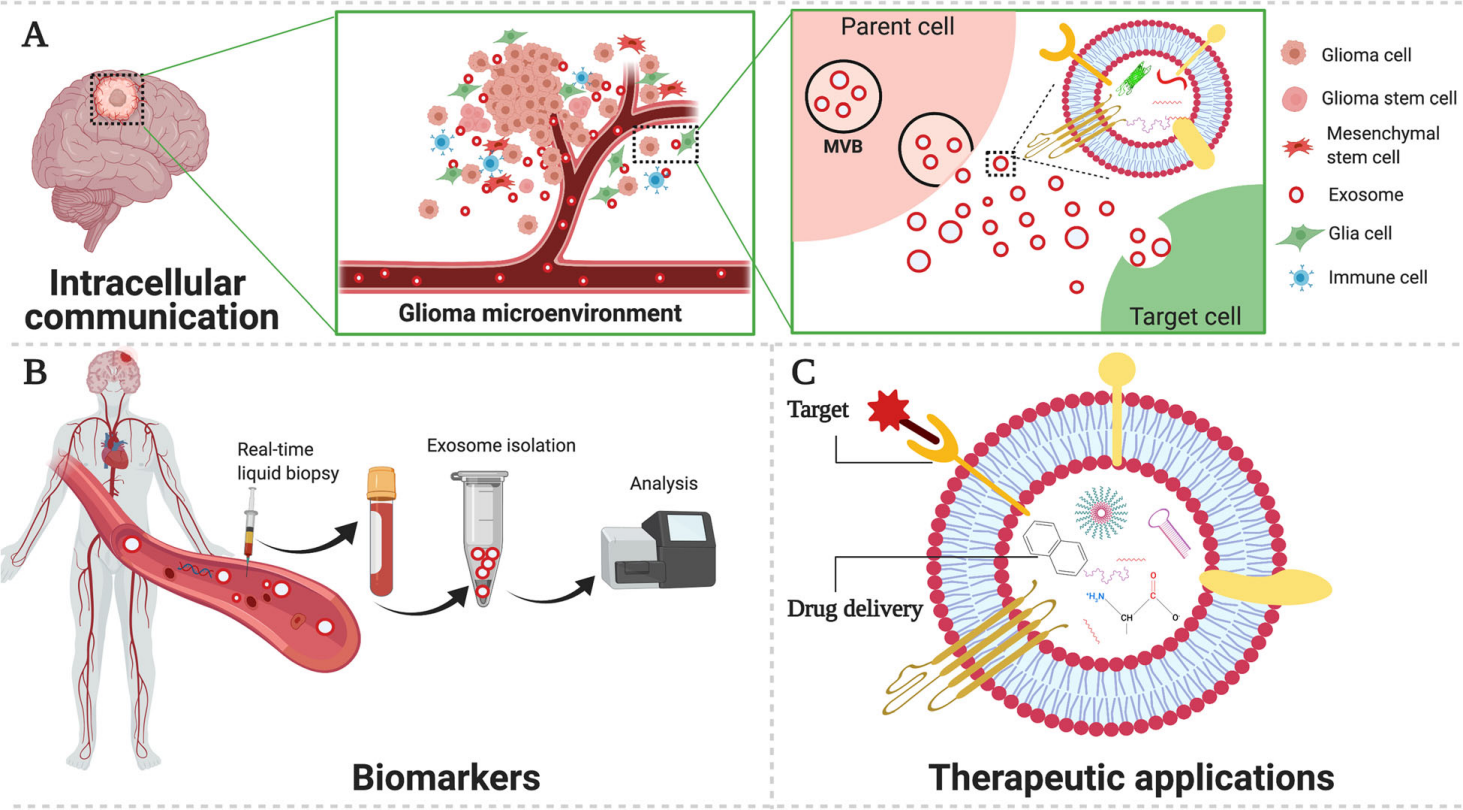
Roles of exosomes in glioma. **a** exosomes participate in cell-to-cell communication by delivering various bioactive molecules, including proteins, lipids and nucleic acids, in the tumor microenvironment. **b** exosomes serve as promising biomarkers. **c** exosomes have novel therapeutic applications

Glioma is a complex and heterogeneous tumor. It not only contains tumor cells, but also has many kinds of nontumoral cell types, such as astrocytes, microglia, endothelial cells and immune cells, which together constitute the complex glioma microenvironment [4]. Increasing evidence revealed that communication between tumor cells and the surrounding components in the glioma microenvironment may directly affect various hallmark features of glioma [4, 5]. For example, tumor cells can change the nonneoplastic astrocytes phenotype by the glioma microenvironment, which in turn promotes glioma growth and invasion [6]. In addition, tumor cells have the ability to survive in the local microenvironment, and eventually changing it to their own advantage via multiple types of cell-cell communication [7]. Elucidating the mechanisms by which glioma cells interact with the tumor microenvironment can uncover multiple potential therapeutic targets for clinical applications. Tumor cells can communicate with neighboring or distant cells through a variety of ways, either by direct cell interactions through membrane receptors and their ligands, or by releasing soluble factors, such as cytokines, chemokines, and metabolites [8]. Recently, exosomes, as a new means of intercellular communication, have drawn much attention due to their ability to carry various bioactive molecules that modulate the activities of recipient cells [9, 10] **(**Fig. 1a). Among these bioactive compositions, noncoding RNAs (ncRNAs) are enriched and stable in exosomes, and have attracted substantial attention due to their regulatory function in the initiation and progression of various cancers [11]. In this review, we summarized current research on the roles of exosomal ncRNAs in glioma progression as well as their potential clinical applications.

### Biogenesis and characteristics of exosome

**Fig. 2:**
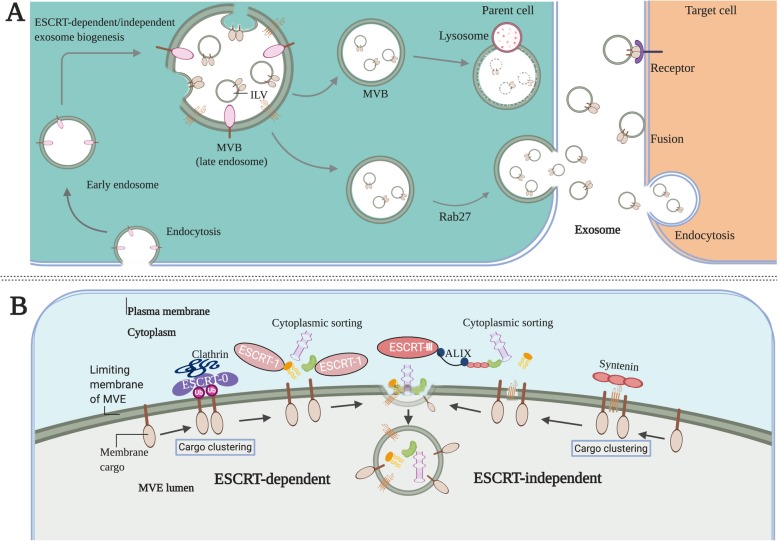
The main process of exosome biogenesis and release. **a** The biogenesis of exosome begins at endosome formation through endocytosis at the plasma membrane, and then early endosomes maturate to multivesicular bodies (MVB). Exosomes are formed as intraluminal vesicles (ILV) in MVBs though endosomal sorting complexes required for transport (ESCRT) dependent or independent pathway. Generally, MVBs either fuse with the lysosome for degradation or fuse with the plasma membrane, which results in ILVs (exosomes) secretion. After secretion, exosomes uptake by target cells is mediated by endocytosis, fusion with the plasma membrane or ligand/receptor interaction. **b** Both the ESCRT- dependent and independent pathway are implicated in controlling the cargos sorting of exosomes

Exosomes, 40–150 nm in diameter, are endosome-derived, small extracellular vesicles (EVs) secreted by a wide variety of cells, which exist in almost all body fluids, including blood, cerebrospinal fluid (CSF), urine, saliva and breast milk [12, 13]. The secretory quantity of exosomes and their contents can vary according to their biogenesis, cell of origin and cell’s status. Studies have shown that some molecules are enriched in exosomes while others are barely present, suggesting a selective sorting feature [14]. The biogenesis of exosome begins at endosome formation through endocytosis at the plasma membrane, and then early endosomes maturate to multivesicular endosomes (MVEs). Exosomes are generated within the endosomal system as intraluminal vesicles (ILVs), which formed by the inward budding of the limiting membrane of MVEs [13] **(**Fig. 2a**)**. The biogenesis of exosome involves a series of sequential molecular machineries, and the most important one is the endosomal sorting complexes required for transport (ESCRT) machinery, which plays essentially role in the formation of ILVs and MVEs as a driver of membrane shaping and scission [13, 15]. The ESCRT system comprises ESCRT-0, −I, −II, and -III, which act in a stepwise manner wherein ESCRT-0 and ESCRT-I participate in cargo clustering and recruitment of ubiquitylated transmembrane cargoes, ESCRT-II takes charge of initiating the inward budding process, and ESCRT-III perform budding and fission of vesicles. The proteins syntenin and ALIX (the ESCRT accessory protein ALG-2 interacting protein X) can intersect with the ESCRT pathway, which regulates the process of exosome biogenesis [16] **(**Fig. 2b**)**. In addition, several studies suggest that exosomes can still be formed despite the depletion of ESCRT complexes, suggesting the existence of an ESCRT-independent pathway [17]. Tetraspanins, specifically CD9, CD63 and CD81, are reported to play important roles in the ESCRT-independent endosomal sorting [13, 18]**.** Thus, both the ESCRT-dependent and -independent pathways are implicated in exosome biogenesis, and their role and contribution may vary depending on the specific cargoes and cell types. Generally, the MVEs either fuse with the lysosome for degradation or fuse with plasma membrane, which secretes the ILVs (exosomes) into the extracellular space **(**Fig. 2a**)**. Like its biogenesis, exosome release also involves several crucial factors and the Rab GTPase family, such as Rab27a and Rab27b, are involved in the trafficking of MVBs to a specific cell membrane region [19]. After secretion, exosomes interact with neighboring or distant recipient cells through several ways, including ligand/receptor interaction, direct membrane fusion and endocytosis, to modulate the activities of recipient cells [20].

The release of exosome into extracellular environment was initially assumed to be a means of eliminating unneeded materials in cells and its biological significance was ignored for a long time [21]. However, extensive research now suggests that exosomes are critical mediators for cell-to-cell communication and involved in the pathogenesis of many diseases, including cancers [22, 23]. Tumor-derived exosomes can change the behavior of surrounding stromal cells, which ultimately create a suitable microenvironment for tumor growth [24]. Meanwhile, stromal cells in the tumor microenvironment can in turn affect tumor progression through the release of exosomes [25–30]. Thus, the complex interactions between tumor and stromal cells via exosomes in local or distant microenvironments promote proliferation, invasion, angiogenesis, immune-escape, metastases and treatment resistance [31]. Furthermore, there is growing interest in the potential clinical application of exosome, with a focus on biomarkers of disease and therapeutic delivery vehicles [12] **(**Fig. 1b and c**).**

### Noncoding RNAs in exosome

A variety of molecules have been identified in exosomes, including proteins, lipids and nucleic acids [32]. The double-layer membranes of exosome protect these molecules from proteases, nucleases, and other environmental impacts [33]. Besides, these molecules in exosomes are selectively packaged, secreted and transferred between cells, and are highly variable depending upon the parental cells and pathophysiological conditions [34]. Increasing evidence has shown that exosomes are enriched with ncRNAs, including microRNA (miRNAs), long-noncoding RNA (lncRNA), circular RNA (circRNA), piwi-interacting RNAs (piRNAs) and tRNA-derived small noncoding RNA (tsRNA), which play important roles in various pathophysiological processes, especially in cancers [35–38]. With the discovery of ncRNAs in exosomes and further research, many new functions and applications have emerged, from new ways of cell-to-cell communication to promising disease biomarkers and, given the biocompatibility of exosomes, possibly novel therapeutic applications. In this review, we mainly focused on the exosomal miRNAs, lncRNAs, and circRNAs in glioma.

#### MicroRNA

**Fig. 3:**
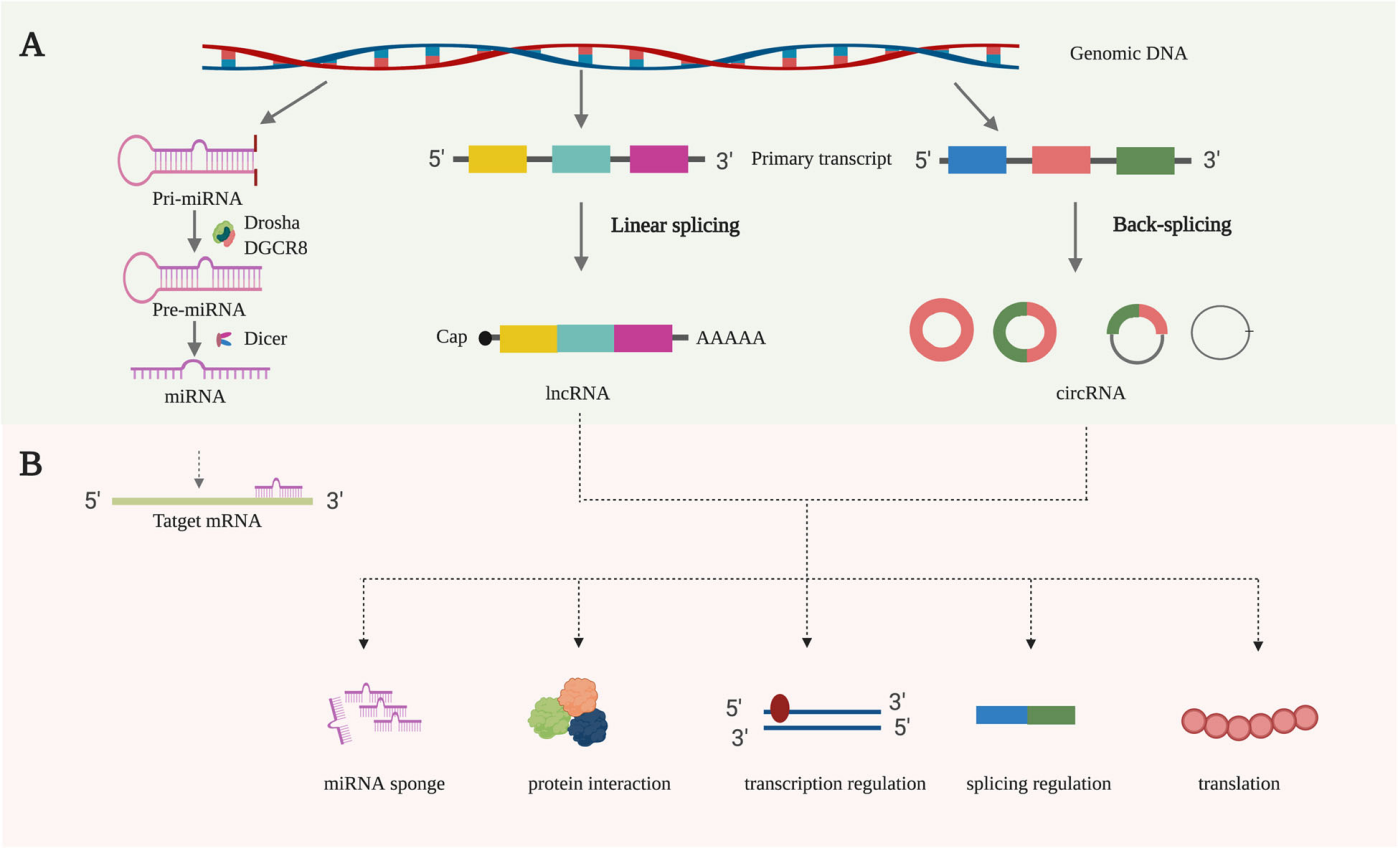
The biogenesis (**a**) and potential functions (**b**) of miRNA, lncRNA and circRNA

MiRNAs are small ncRNAs consisting of approximately 21–25 nucleotides, which act as regulators of gene expression by complementary binding of targeted mRNA’s 3′ untranslated regions (UTR), thus reducing the stability or inhibiting translation of target genes [39] **(**Fig. 3b**)**. Each miRNA may have multiple target mRNAs, and one mRNA may be targeted by several miRNAs, thus leading to a complex regulatory network. The biogenesis of miRNA begins with transcribing miRNA gene into large primary transcript (pri-miRNA), and it is then cleaved into a ~ 85-nucleotide stem–loop structure called precursor miRNA (pre-miRNA) by the microprocessor complex including the type III RNase Drosha and the RNA-binding protein DGCR8. The pre-miRNA is then processed to a ~ 20–22-nucleotide miRNA/miRNA duplex by the RNase III enzyme Dicer. These miRNA duplexes are unstable and soon are cleaved into single stranded mature miRNAs. The mature miRNA is loaded onto an Argonaute protein (AGO) to form the RNA induced silencing complex (RISC), which then binds to target mRNA for post-transcriptional gene silencing [39] **(**Fig. 3a**)**. Currently, the latest release of the miRBase database (v22) contains 1917 annotated hairpin precursors, and 2654 mature miRNAs in the human genome [40]. Numerous studies have indicated that dysregulation of miRNA expression is closely associated with many human diseases, including cancers. MiRNAs may play oncogenic roles or function as tumor suppressors under certain conditions [41]. Recently, the discovery of miRNAs in exosomes gained increasing attention. Extensive studies suggest that miRNAs are selectively sorted into exosomes that participate in cell-to-cell communication in the tumor microenvironment and play an essential part in tumor biology [5]. Furthermore, the easy access, abundance and stability of exosomal miRNAs in biofluids have made them ideal biomarkers for various types of cancers, including gliomas [42].

#### Long-noncoding RNA

LncRNAs are defined as transcripts longer than 200 nucleotides in length that have no or limited protein-coding capacity [43]. LncRNAs exist in either the nucleus or cytoplasm, and play distinct functions depend on their subcellular localizations. In the nucleus, lncRNAs may participate in transcriptional regulation of gene expression and mRNA splicing. While in the cytoplasm, they could affect mRNA stability and regulate protein function [44]. Moreover, lncRNAs exert their functions through multiple molecular mechanisms, such as binding with DNA to modulate gene transcription, acting as the competing endogenous RNA (ceRNA) or miRNA sponges to regulate gene expression at post-transcriptional level, associating with proteins, and encoding functional small peptides [45] **(**Fig. 3b**)**. Over the last decade, it has been increasingly demonstrated that lncRNAs are involve in many pathophysiological processes, such as cell cycle regulation, cell differentiation, and innate immune response [45, 46]. Furthermore, dysregulation of lncRNAs expression has been observed in many human cancers, including gliomas, and is closely related to the stage and grade of cancers through their roles as tumor suppressors or oncogenes [43]. Interestingly, lncRNAs can also be selectively sorted into exosomes and contribute to intercellular communication in the tumor microenvironment [47]. Exosomal lncRNAs can participate in the onset and progression of gliomas, such as proliferation, invasion, angiogenesis and drug resistance, which may act as attractive therapeutic targets [6, 48, 49]. Furthermore, exosomal lncRNAs have potential for diagnostic and prognostic biomarkers [47].

#### Circular RNA

CircRNAs, a novel class of endogenous ncRNAs with a closed loop structure, are primarily produced from pre-mRNAs via back-splicing [50] **(**Fig. 3a**)**. CircRNAs were initially thought to be byproducts of RNA splicing. Recently, researchers show great interests in the properties and functions of circRNAs because of the discovery of more circRNAs with important functions [51]. Furthermore, circRNAs exhibit tissue- and developmental stage-specific expression, and their expression is often independent of related linear isoforms [52]. Currently, several studies have demonstrated that circRNA expression is most abundant in the brain compared with other tissues, implying their important roles in brain function and brain diseases [53]. Though the exact functions of most circRNAs remain unclear, accumulating evidence suggests that circRNAs may regulate gene expression at multiple levels, such as regulating transcription of their parental genes, affecting splicing of their linear cognates, acting as miRNA sponges, regulating the function of RNA-binding proteins, and encoding peptides [52, 54] **(**Fig. 3b**)**. CircRNAs are reported to participate in various physiological and pathological processes, particularly in cancers [51, 55]. Many studies have shown that circRNAs are aberrantly expressed in cancer including gliomas and play a vital role in tumor initiation and progression [56, 57].

Recently, the presence of circRNAs, in addition to miRNAs and lncRNAs, has also been detected in exosomes by Li and colleagues in 2015 [58]. Research on exosomal circRNAs is still at an initial stage, but a small number of studies suggest that circRNAs are enriched in exosomes, through which circRNAs start their circulation and reach the recipient cells, thus carrying out their various biological functions [58, 59]. Unlike linear RNAs, circRNAs with loop structure are much more stable and have a longer half-life, therefore, making them more suitable for developing biomarkers than the linear RNA transcripts [57, 60].

### The functional roles of exosomal ncRNAs in glioma

As the most common and deadly primary brain tumors, gliomas typically exhibit as rapid proliferation and extensive invasion, immune surveillance escape, angiogenesis induction, and drug resistant. However, their underlying molecular mechanisms remain unclear yet. Emerging evidence suggests that exosomes mediate glioma initiation and progression through transferring bioactive molecules between different cell populations [61]. As one of important contents, exosomal ncRNAs play key roles in multi-aspects of tumor biology, including proliferation, invasion, angiogenesis, immune-escape, metastases and treatment resistance within the tumor microenvironment [11]. In the following sections, we summarized the current research on the specific roles and mechanisms of exosomal ncRNAs in glioma progression **(**Table 1**)**.

**Table 1:** The biological functions of exosomal ncRNAs in glioma

NcRNAs	Parent cell	Target cell	Biological function	Reference
miR-221	U87MG	SHG-44	Promote proliferation, migration and TMZ resistance	[62]
lncRNA-ATB	Glioma cells	Normal human astrocytes	Promote invasion	[6]
miR-148a	Glioma cells	Glioma cells	Promote proliferation and metastasis	[63]
miR-451/miR-21	Glioma cells	Microglia/macrophages	Promote proliferation and immune suppression	[24]
miR-1587	Mesenchymal Stem Cells	Glioma Stem-like Cells	Increase tumorigenicity	[64]
miR-1	Glioblastoma cells	Endothelial cells and glioblastoma cells	Inhibit angiogenesis, invasion, and neurosphere formation	[65]
miR-124	Mesenchymal stem cells	glioblastoma cells	Inhibit proliferation, migration and confer chemosensitivity	[28]
miR-302-367	Glioma stem-like cells	Glioblastoma cells	Inhibit growth	[66]
miR-7	Mesenchymal stem cells	Glioblastoma cells	Increase apoptosis and suppress growth	[30]
miR-584	Mesenchymal stem cells	Glioma cells	Suppress tumor progress	[29]
miR-146b	Marrow stromal cells	Gliosarcoma cells	Reduce glioma growth in vivo	[25]
miR-124a	Mesenchymal stem cells	Glioma stem cell	Antiglioma agent	[31]
miRNA-199a	Mesenchymal stem cells	Glioma cells	Inhibit proliferation, invasion and enhance chemosensitivity	[27]
miR-375	Marrow stromal cells	Glioma cells	Inhibit glioma progression	[26]
lncRNA-HOTAIR	Glioblastoma cells	Endothelial cells	Promote angiogenesis	[49]
lincRNA-CCAT2	Glioma cells	Endothelial cells	Promote angiogenesis	[67]
lincRNA-POU3F3	Glioma cells	Endothelial cells	Promote angiogenesis	[68]
miR- 26a	Glioma stem cells	Endothelial cells	Promote angiogenesis	[69]
miR-21	Glioma stem cells	Endothelial cells	Promotes angiogenesis	[70]
miR-9	Glioma cells	Endothelial cells	Promote tumorigenesis and angiogenesis	[71]
miR-10a, miR-21	Hypoxic glioma cells	Myeloid-derived suppressor cells	Mediate immunosuppressive microenvironments	[72]
miR-29a, miR-92a	Hypoxic glioma cells	Myeloid-derived suppressor cells	Mediate immunosuppressive microenvironments	[73]
miR-1246	Hypoxic glioma cells	Macrophages	Mediate immunosuppressive microenvironment	[74]
miR-21	Glioma cells	Microglia	Mediate immunosuppressive microenvironment	[75]
miR-151a	TMZ-resistant glioblastoma cells	TMZ-sensitive glioblastoma cells	Enhances chemosensitivity to TMZ	[76]
lncRNA-SBF2-AS1	TMZ-resistant glioblastoma cells	Chemoresponsive glioblastoma cells	Enhances chemoresistance to TMZ	[77]
circATP8B4	Radioresistant glioma cells	Normal glioma cells	Promote cell radioresistance	[78]
miR-301a	Hypoxic glioma cells	Normoxia-cultured glioma cells	Promote radiation resistance	[79]
lncRNA-AHIF	Radioresistant glioblastoma cells	Glioblastoma cells	Promote invasion and radioresistance	[48]

Abbrevations: *TMZ* temozolomide

#### Exosomal ncRNAs and cell proliferation/invasion in glioma

**Fig. 4:**
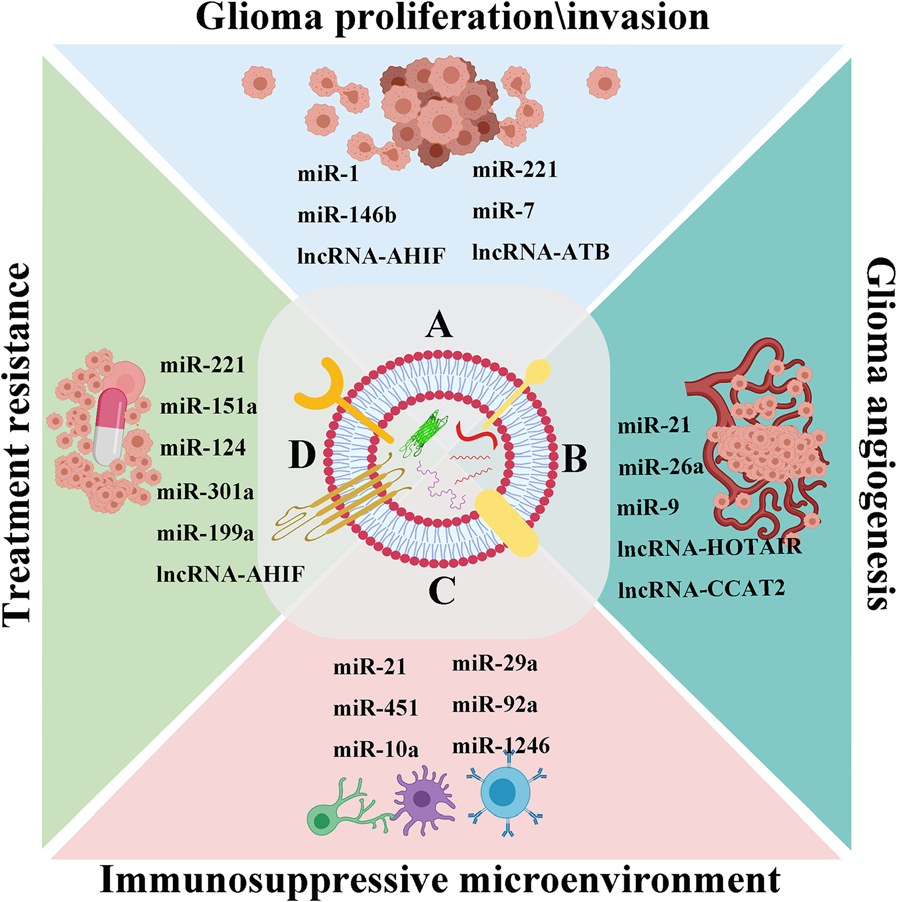
Exosomal ncRNAs play important roles in regulating glioma proliferation/invasion (**a**), angiogenesis (**b**), immune-escape (**c**), and treatment resistance (**d**)

Rapid proliferation and extensive invasion are the most important hallmarks of malignant gliomas, which involve in complex molecular pathways and dynamic crosstalk between tumor cells and microenvironment. These growth features may ultimately lead to the incomplete surgical resection and inevitable tumor recurrence [80]. Hence, identifying the key molecules that associate with tumor proliferation and invasion is critical to devising new treatment strategies. Increasing evidence shows that ncRNAs play important roles in glioma progression [35]. Glioma-derived exosomes carrying a broad range of ncRNAs are reported to exert significant functions in glioma proliferation and invasion through regulating intercellular communication in local and distant microenvironments [6, 48, 63–65, 71] **(**Fig. 4a**)**.

Certain miRNAs are selectively packaged into exosomes and regulate cell proliferation and invasion in glioma [65, 66]. MiR-1 is known as a tumor suppressor in several cancers [81]. Bronisz et al. found that miR-1 could be transferred by GBM–derived EVs to the surrounding GBM cells, and reduced GBM growth and invasion through directly targeting the expression of Annexin A2 (ANXA2), an important pro-oncogenic factor in GBM [65]. Another study by Cai et al. observed an increased level of exosomal miR-148a in GBM patient’s serum when comparing to healthy volunteers. Moreover, miR-148a delivered by exosomes promotes GBM cell proliferation and metastasis via targeting cell adhesion molecule 1 (CADM1) to enhance STAT3 signaling activity. CADM1 was proved to be a tumor suppressor which was downregulated in glioma tissues and most glioma cell lines [63].

Besides miRNAs, some exosomal lncRNAs, such as lncRNA-ATB [6] and lncRNA-AHIF [48], were reported to regulate cell proliferation and invasion in glioma. Bian and colleagues found that glioma cell-derived exosomes had the ability to shuttle lncRNA-ATB to astrocytes and promoted the activation of astrocytes, which could in turn promote the invasion and migration of glioma cells. More importantly, lncRNA-ATB activats astrocytes through the suppression of miR-204-3p in an Argonaute 2 (Ago2)-dependent manner [6]. Taken together, these studies suggest that ncRNAs selectively secreted and transferred between cells in exosomes play important roles in regulating glioma proliferation and invasion.

#### Exosomal ncRNAs and glioma angiogenesis

As a crucial hallmark of blood vessel-rich tumors, angiogenesis plays a significant role in the development and progression of gliomas, especially the GBM [82]. Tumor angiogenesis is a complex process comprising multiple steps, such as degradation of basement membrane, endothelial cell proliferation, migration, sprouting, branching, and tube formation, and depending on the coordination of many regulators [83, 84]. Vascular supply is the fundamental requirement for proliferation and invasion of glioma, for rapid growth of tumor requires even more nutrients and oxygen supply. As relevant studies demonstrated that solid tumors can’t grow larger than 2–3 mm in diameter without inducing their own blood supply [85]. The mechanism underlying angiogenesis is not entirely clear. Aberrant activation of the HIF-1α/VEGF pathway can induce endothelial cell proliferation and migration, which is considered to be one of the underlying causes of angiogenesis [86]. Therefore, a better understanding of the cellular and molecular mechanisms involved in tumor angiogenesis will be required for developing novel anti-angiogenic therapies.

Extensive studies have revealed that ncRNAs play important regulatory roles in tumor angiogenesis [84]. In the tumor microenvironment, exosomes released by different cell types, such as tumor cells, mesenchymal stem cells and stromal cells, can be captured by endothelial cells that encourage growth of new blood vessels [68–70]. Increasing evidence suggests that exosomes exert the function of angiogenesis regulation partly by delivering ncRNAs in the glioma microenvironment [49, 67–71] **(**Fig. 4b**)**. As an oncomiR, miR-21 is expressed in a wide range of cancers, and regulates many biological processes [75]. Sun et al. found that glioma stem cells-derived exosomes carrying miR-21 could promote the angiogenic ability of endothelial cells through upregulating VEGF expression, which further interacting with VEGFR2 to activate downstream PI3-kinase/Akt pathway [70]. Furthermore, Chen and colleagues revealed that miR-9 could be secreted from glioma cells via exosomes and was then absorbed by vascular endothelial cells. MiR-9 could target PHD3, which mediated HIF-1α/VEGF signaling pathway to increase glioma angiogenesis. This study suggested miR-9 could be treated as a potential therapeutic target for glioma [71]. There are many exosomal lncRNAs, such as lncRNA-HOTAIR, lincRNA-CCAT2 and lincRNA-POU3F3, that have been confirmed to play crucial roles in the regulation of glioma angiogenesis [49, 67, 68]. For instance, the lncRNA-HOTAIR, a well-characterized oncogene in various cancers, including gliomas, is associated with tumor proliferation, migration and invasion, and its expression is closely associated with glioma grades [87]. Ma et al. found that HOTAIR can be secreted by glioma cells via exosomes and delivered to endothelial cells, thus promoting glioma angiogenesis though regulation of VEGF expression [49]. Similarly, another study by Lang et al. revealed that glioma cells could transfer linc-CCAT2 to endothelial cells by exosomes to activate VEGFA and TGFβ, and thereby promoting angiogenesis [67].

Glioma stem cells (GSCs) are a small minority of the cancer cell population that show multipotent glioma-initiating capacity [88]. Increasing evidence suggests that GSCs are found in close association with vascular niches [89], while blood vessels support GSCs, and these stem cells in turn may regulate and contribute to the glioma angiogenesis through various ways, including the secretion of exosomes. Wang et al. also found GSCs could release exosomes that mediate cellular communication by delivering miRNAs. The results showed that GSCs-derived exosomes overexpressing miR-26a contributed to enhanced proliferation and angiogenesis of human brain microvascular endothelial cells in vitro through specifically binding to PTEN, which further regulated the PI3K/Akt signaling pathway in glioma [69]. These studies suggested that GSCs can promote the angiogenic ability of endothelial cells via exosomal ncRNAs.

#### Exosomal ncRNAs and immunosuppressive microenvironment

As a substantial part of the glioma microenvironment, glioma-associated immune cells have an emerging role in tumor progression and in controlling anti-tumor immune responses [4, 90]. Bone marrow-derived macrophages, along with the resident CNS microglia, can constitute up to 30% of the tumor mass, which exert pro-tumorigenic functions and creat an immunosuppressive microenvironment [4, 91]. Glioma cells can shape the immune cells via exosomes, thus creating an optimum microenvironment for tumor survival and growth [23, 61]. Glioma-associated microglia and macrophages are key drivers of the local immunosuppression promoting tumor progression and its resistance to immunomodulating therapeutic strategies [92]. Hence, targeting these immune cells and related moleculars represents a novel promising treatment strategy for glioma patients.

Recent studies revealed that glioma cells derived exosomal ncRNAs, such as miR-451 [24], miR-1246 [74] and miR-21 [75], could change the phenotype of microglia and macrophages to promote glioma growth and survival **(**Fig. 4c**)**. Van der Vos and colleagues investigated exosomal miRNA released from glioma cells and taken up by microglia and macrophages as a means by which tumor cells manipulate normal cells in glioma microenvironment [24]. They further monitored phenotypic changes in microglia exposed to isolated GBM derived EVs in culture, as well as their effects on target mRNAs in an intracranial mouse glioma model. Their results showed that high levels of miR-451/miR-21 were transferred from glioma cells to microglia via EVs with a decrease of a common mRNA target encoding c-Myc, which led to increased microglia proliferation and shifting of cytokine profile toward immune suppression [24]. Myeloid-derived suppressor cells (MDSCs) play an important role in mediating the formation of an immunosuppressive environment and assisting gliomas in evading the host immune response, but the specific mechanisms have not been clearly defined. Guo et al. isolated normoxia-stimulated and hypoxia-stimulated glioma-derived exosomes, which could be taken up by MDSCs, and studied their MDSC induction abilities in vivo and in vitro. The authors found that hypoxia- stimulated glioma-derived exosomes had a stronger ability to induce MDSCs, and it was the hypoxia-inducible expression of exosomal miR-10a and miR-21 that mediated MDSC differentiation and activation by targeting RAR-related orphan receptor alpha (RORA) and phosphatase and tensin homolog (PTEN) [72]. In another study by the same authors found glioma exosomal miR-29a/miR-92a could also mediate the formation of immunosuppressive microenvironment via enhancing the proliferation and differentiation of functional MDSCs by targeting high-mobility group box transcription factor 1 (Hbp1) and protein kinase cAMP-dependent type I regulatory subunit alpha (Prkar1a), respectively [73]. These studies suggest that glioma can influence the differentiation and activation of MDSCs via exosomal miRNAs, thus affecting the entirety of tumor immune environment.

#### Exosomal ncRNAs and treatment resistance

For high-grade gliomas, radiotherapy and chemotherapy after maximum safe resection have always been the cornerstone of treatment [2]. However, it’s reported that high-grade gliomas, especially GBM, tend to exhibit advanced resistance to multiple anti-tumor therapeutic methods [93]. Radio- and chemoresistance in glioma are major factors affecting the effectiveness of treatment and leading to poor prognosis. There is compelling evidence that GSCs are resistant to radiation and chemotherapy, and thereby form the basis for glioma recurrence [94]. In recent years, accumulating evidences suggest that exosomes, as an important communicator of intercellular communication, may led to horizontal propagation of resistance capacity between heterogeneous populations of cancer cells, and ultimately blocking the successful treatment of many cancers [11, 14]. In addition, recent studies revealed that ncRNAs delivered by exosomes play important roles in promoting tumor cell adaptation to the tumor microenvironment and treatment resistance [62, 95] **(**Fig. 4d**)**. Thus, exploring the mechanism of treatment resistance can be extremely significant in order to eradicate these incurable tumors.

As a monofunctional DNA-alkylating agent, temozolomide (TMZ) is used as a first-line chemotherapy in glioma through inducing DNA damage in glioma cells [96]. TMZ resistance limits the durability of the treatment response and affects the prognosis of patients. Relevant studies suggest that TMZ-resistant glioma cell-derived exosomes can transfer TMZ chemoresistance to recipient TMZ-sensitive cells via exosomes [76, 77]. Zhang et al. found that lncRNA SBF2-AS1 showed upregulated expression in TMZ-resistant GBM cells and glioma tissues, and was associated with TMZ resistance. Meanwhile, exosomal could transfer lncRNA SBF2-AS1 from chemoresistant GBM cells to chemoresponsive GBM cells to spread TMZ resistance. Further study revealed lncRNA SBF2-AS1 functioned as a ceRNA and could regulate the expression of X-ray repair cross complementing 4 (XRCC4) by sponging miR-151a-3p, thus promoting the repair process of TMZ-induced DNA damage [77].

Exosomal ncRNAs released by glioma cells can also interfere with the sensitivity to radiotherapy and thus affect the treatment efficiency. Yang et al. reported that exosomal miR-301a specifically secreted by hypoxic GBM cells could transfer to corresponding normoxia-cultured cells and promote radiation resistance by targeting TCEAL7 genes [79]. In addition, a recent study demonstrated that circRNA-ATP8B4 from radioresistant GBM derived EVs could also promote glioma radioresistance by sponging miR-766 [78]. Therefore, these exosomal ncRNAs harbor a great therapeutic significance as promising agents for future clinical applications either independently or complementary to modulate radio- and chemoresistance in glioma treatment.

### Potential clinical applications of exosomal ncRNAs in glioma

#### Exosomal ncRNAs serve as promising biomarkers

Nowadays, advanced neuroimaging, such as magnetic resonance imaging (MRI), is the principal diagnostic method for patients with suspected brain lesions. Although neuroimaging may suggest glioma diagnosis, other brain lesions might share quite similar radiological features, which makes it difficult for differential diagnosis [97]. Furthermore, it is often difficult to distinguish true radiographic progression from pseudoprogression in the postoperative follow-up [98]. Moreover, the commonly used high-resolution MRI, which by the way, isn’t very high resolution compared to histology examination. The lowest MRI resolution ranges on the order of millimeters, whereas the dimensions of the tumor cell are in micrometers [99], this disparity in scale may lead to delay in diagnosis and treatment. Histological examination of the tumor tissue obtained by biopsy or surgery is currently the golden standard for definite diagnosis. However, these practices may be associated with significant morbidity, and repeated sampling of tumor tissue is not always appropriate. Besides, biopsies may not reflect the heterogeneity of gliomas due to the small amounts of tissue samples. As mentioned earlier, TMZ is used as a first-line chemotherapy in high-grade glioma after maximum safe resection [96]. TMZ resistance limits the durability of the treatment response. Hence, it will be important to monitor TMZ resistance in biofluids for adjusting treatments. Above all, developing of minimal invasive methods, for the detection, differentiation and monitoring of gliomas will be clinically meaningful, especially in those patients in which surgery is contraindicated or biopsy results are inconclusive.

Although conventional strategies for liquid biopsies have been shown to be promising, it remains a huge challenge to develop clinically validated tumor detection biomarkers, especially for gliomas. At present, biomarkers which have been studied extensively, including circulating tumor cells (CTCs), circulating tumor DNA (ctDNA), circulating proteins, and EVs [100]. With the perspective of various types of circulating biomarkers, the primary concern is how representative they are of the whole tumor, or at least they can reflect the primary biological features of the tumor. Ideally, CTCs could enable profiling of the whole-tumor genome, however the number of CTCs is too small to detect, and can only reflect a single cell type of the heterogeneous tumor. Furthermore, the ctDNA contains the mutations present in tumor and seems to be much more abundant than CTCs, but it also fails to reflect tumor heterogeneity. Whereas, exosomes contain a variety of functional molecules that can reflect the complex heterogeneity of the whole tumor. And, best of all, they are extremely stable and are readily accessible in nearly all types of human biofluids [101]. These factors make exosomes the most promising biomarkers.

An increasing number of studies have evidenced key roles of ncRNAs in the initiation and progression of various cancers, including gliomas [102]. In addition, ncRNAs are secreted into the circulation either circulate freely or be selectively packaged into exosomes, suggesting these molecules can be used as new biomarkers. The double-layer lipid membrane of exosomes can protect ncRNAs from the ribonuclease-mediated degradation. In addition, previous research has suggested that exosomes can cross the blood brain barrier (BBB) [103]. Hence, the exosomal ncRNAs have a good clinical application prospect in glioma diagnosis and caused more and more attention [104–108] **(**Table 2**)**.

**Table 2:** The biomarker potential of exosomal ncRNAs in glioma

NcRNA Canditates	Source of exosome	Exosome isolation techniques	System	Biomarker potential	Reference
miR-21	CSF	Ultracentrifugation	GBM	A biomarker for GBM diagnosis	[109]
miR-21	CSF & serum	Ultracentrifugation	Glioma	A biomarker for glioma diagnosis and prognosis	[110]
miR-151a	CSF & serum	Ultracentrifugation	GBM	Predict prognosis of GBM patients	[76]
miR-320 & miR-574-3p	Serum	Precipitation method	GBM	A biomarker for GBM diagnosis	[108]
miR-221	Serum	Precipitation method	Glioma	A biomarker for glioma diagnosis and grade prediction	[62]
miR-21, miR-222 & miR-124-3p	Serum	PEG-based method	Glioma	A biomarker for glioma diagnosis and grade prediction	[104]
miR-301a	Serum	Precipitation method	Glioma	Serve as diagnostic and prognostic biomarker	[105]
miR-148a	Serum	Precipitation method	GBM	A biomarker for GBM diagnosis	[63]
miR-454-3p	Serum	Immunoaffinity capture	Glioma	A biomarker for glioma diagnosis	[106]
lncRNA-HOTAIR	Serum	Precipitation method	GBM	A biomarker for GBM diagnosis	[107]
lncRNA-SBF2-AS1	Serum	Ultracentrifugation	GBM	Serve as diagnostic biomarker for therapy-refractory GBM	[77]

Abbrevations: *CSF* cerebrospinal fluid, *GBM* glioblastoma, *PEG* polyethylene glycol

CSF examination has been widely used in clinical practice for monitoring of CNS diseases but is less used in gliomas to date. Previous studies suggested that the miR-21 expression in glioma was up-regulated, and the levels of miR-21 were also correlated with the histological grade [110, 111]. Hence, miR-21 has drawn great attentions for its potential as diagnostic and prognostic biomarkers. Akers et al. found that the CSF EV miR-21 levels from GBM patients were, on average, ten-fold higher than that isolated from controls, which suggested CSF EV miR-21 may serve as a diagnostic biomarker for GBM patients [109]. A similar study by shi et al. also found that exosomal miR-21 levels in the CSF of glioma patients were significantly higher than in the controls. Interestingly, the results showed no difference in serum-derived exosomal miR-21 expression. In addition, CSF exosomal miR-21 levels were correlated with tumor recurrence and spinal/ventricle metastasis [110]. These studies suggest CSF-derived exosomal ncRNAs could be used as promising biomarkers for glioma diagnosis and prognosis.

Another important source of glioma biomarker is blood. Yang et al. found that the miR-221 expression level was increased in high-grade glioma tissue, and further analysis revealed a significant higher serum exosomal miR-221 level in glioma patients than in controls. More importantly, exosomal miR-221 level in serum increased with the glioma grades [62]. This study suggests that exosomal miR-221 may serve as a novel diagnostic biomarker for glioma patients. Information about long-term prognosis of glioma patients may assist identification of patients requiring special attention. With the increased availability of various types of predictive biomarkers, a non-invasive, rapid and highly sensitive biomarker that can evaluate the patient’s prognosis is clinically meaningful. Recent work has shown that exosomal ncRNAs may serve as independent prognostic parameters for gliomas [105]. Lan and colleagues found that an increased serum exosomal miR-301a level was associated with a longer overall survival (OS). And further Cox regression analyses verified serum exosomal miR-301a level was independently associated with patient’s OS [105]. Acquired drug resistance is a major constraining factor in clinical treatment of glioma. Thus, identifying the underlying drug resistant patients and finding alternative methods of treatment timely are extremely important to improve prognosis. Zhang et al. found that higher serum exosomal lncRNA SBF2-AS1 levels from recurrent GBM patients were associated with worse prognosis and poorer response to TMZ treatment [77]. In conclusion, these studies suggest that exosomal ncRNAs in biofluids have shown great potential in biomarker development, and their clinical applications deserve further research.

#### The potential application of exosomal ncRNAs in anti-glioma therapy

Considering the important biological functions of exosomal ncRNAs in gliomas, the strategies of specifically targeting exosomes or their cargoes, such as the ncRNAs, can be promising therapeutic options in the treatment of gliomas. Hence, many ongoing studies are designed either to modulate exosomes production or block exosomes uptake pathways so as to achieve the goal of treating cancer [112]. One study proposed a novel device strategy that allowed rapid extracorporeal capture and selective retention of target particles < 200 nm from the entire circulatory system using an affinity plasmapheresis platform known as the Aethlon ADAPTTM (adaptive dialysis-like affinity platform technology) system, which can be used to incorporate diverse affinity agents for capturing cancer-specific exosomes on the basis of displaying surface proteins or glycoproteins [113]. The safety and effectiveness of this strategy are supported by clinical experience in hepatitis C virus (HCV)-infected patients using an ADAPTTM device to reduce the systemic load of virions, which have similar sizes and glycosylated surfaces as cancer-derived exosomes [113, 114]. Therefore, the concept of reducing circulating cancer-specific exosomes could offer a unique treatment for cancer patients. NcRNAs, such as miRNAs, lncRNAs and circRNAs, are important regulatory molecules in the initiation and development of gliomas, and play crucial roles in almost every aspect of biological processes, including tumor proliferation\invasion, angiogenesis and treatment resistance [35]. With advances in the understanding of ncRNA function, exosomal ncRNAs will become attractive therapeutic targets for the treatment of gliomas.

The BBB helps protect the brain, but it also creates difficulties in treating neurological disorders, including gliomas [115]. As double-layer phospholipid membrane vesicles with abundant bioactive molecules inside, exosomes exhibit distinct advantages as gene therapy delivery vectors. Furthermore, exosomes have their own special features, such as small size, flexibility and good histocompatibility, which enable them to cross the major biological barriers in the body such as the BBB [103]. Lai et al. designed a highly sensitive EVs reporter system, which enables tracking of EVs biodistribution over time in vivo imaging. Through this system, they found that systemically injected EVs can be delivered to tumor sites within an hour [116]. Therefore, exosomes could be used as a promising therapeutic delivery system in glioma treatment.

As exosomes serve as natural ncRNA carriers, they may be adapted to deliver tumor suppressive ncRNAs. Generally, tumor suppressive ncRNAs are downregulated in cancer, and overexpressing these ncRNAs via exosomes in tumor cells may inhibit tumor progression. Katakowski and colleagues found that intra-tumor injection of exosomes, which were released by MSCs overexpressing miR-146b, an anti-glioma miRNA that can reduce glioma cell invasion, migration and viability, significantly reduced glioma xenograft growth in a rat model of primary brain tumor [25]. A similar study by Fareh et al. engineered primary glioma cells to stably express the miR-302-367, and this tumor suppressive miRNA was largely enclosed in exosomes, which were internalized by the neighboring GBM cells, thus inducing antitumor cell effects in vitro and in vivo [66]. Furthermore, Munoz et al. found that delivery of functional anti-miR-9 by MSCs–derived exosomes to GBM cells could make GBM sensitive to TMZ [117]. These studies suggest that exosomes could be used as vehicles for delivery of anti-tumor ncRNAs, demonstrating their potential use for therapeutic applications. In addition to transfering various ncRNAs, other types of both endogenous and exogenous therapeutic cargoes such as proteins, lipophilic small molecules and drugs, can also be loaded into these particles for cancer therapy [112]. However, a vast amount of further study is needed before these therapeutic approaches would be available for clinical application.

## Conclusions and prospects

Glioma is the most common malignant intracranial tumor with poor prognosis and could be resistant to various therapies. The pathogenesis of this deadly disease is not entirely clear which may involve thousands of genes, signals and molecular components. NcRNAs including miRNA, lncRNA and circRNA, account for a large portion of human transcriptome and play indispensable roles in the glioma initiation and progression. Exosomes represent a new means of intercellular communication and are enriched with ncRNA content. Extensive studies suggest that exosomal ncRNAs are selectively packaged, secreted and transferred between cells that participate in cell-to-cell communication in the tumor microenvironment, and can modulate numerous hallmarks of glioma, such as proliferation, invasion, angiogenesis, immune-escape, and treatment resistance. In addition, the discovery that exosomes carry various bioactive molecules and can readily cross biological barriers (for example, BBB) hold great promises for clinical applications, including diagnostics and therapy.

Though promising, there is still a long way to go before the exosomal ncRNA can be used in clinical practice. Exosomes in the body fluids are often a heterogeneous population of vesicles of unknown origin. Accordingly, identifying and isolating the tumor-specific exosomes is particularly important, however, this could be hard to achieve currently, though there are multiple methods for isolation with the pros and cons of each. Furthermore, given the limited amount of genetic materials present in biofluid exosomes, the maximum use, normalization and quantitation remain urgent questions to solve. Exosomes mediate intercellular communication through specifically and selectively transferring bioactive molecules between cells. However, how molecules are synthesized and selectively packaged into exosomes is largely unknown, and further studies are needed to reveal the detail mechanisms. Exosomes could be engineered to deliver therapeutic factors, such as RNA, proteins and drugs, to target cells for therapeutic applications. But a precondition is that exosomes must be able to accurately find its target and release its cargo. However, the molecular mechanisms that target exosomes to a given cell type or tissue are far from clear. With progressively better understanding of these unknowns, we believe exosomes will become increasingly valuable agents in the diagnosis and therapy of diseases in the near future.

## Data Availability

Not applicable.

## Abbreviations

AGO: Argonaute protein
ALIX: ALG-2 interacting protein X
BBB: Blood brain barrier
CADM1: Cell adhesion molecule 1
ceRNA: Competing endogenous RNA
circRNA: Circular RNA
CNS: Central nervous system
CSF: Cerebrospinal fluid
ctc: Circulating tumor cell
ctDNA: Circulating tumor DNA
ESCRT: Endosomal sorting complexes required for transport
EV: Extracellular vesicle
GBM: Glioblastoma
GSC: Glioma stem cell
ILV: Intraluminal vesicle
lncRNA: Long-noncoding RNA
MDSC: Myeloid-derived suppressor cell
miRNA: microRNA
MRI: Magnetic resonance imaging
MVE: Multivesicular endosome
ncRNA: Noncoding RNA
OS: Overall survival
piRNA: Piwi-interacting RNA
RISC: RNA induced silencing complex
TMZ: Temozolomide
tsRNA: tRNA-derived small noncoding RNA
UTR: Untranslated regions
VEGF: Vascular endothelial growth factor
